# Dr. Malcolm Ney McNaughton

**Published:** 1912-08

**Authors:** 


Dr. Malcolm Ney McNaughton, Buffalo, 1868, died at Villisca, Ill., June 23, aged 63. Dr. McNaughton was a good example of the negative inference from the old adage that a rolling stone gathers no moss. He began practice in Villisca almost immediately after his graduation and, for some time before his death, was president of the First National Bank.

